# Hemato-biochemical alterations and urinalysis in dogs suffering from benign prostatic hyperplasia

**DOI:** 10.14202/vetworld.2017.331-335

**Published:** 2017-03-19

**Authors:** M. R. Das, R. C. Patra, R. K. Das, P. K. Rath, B. P. Mishra

**Affiliations:** 1 Department of Veterinary Clinical Medicine, College of Veterinary Sciences and Animal Husbandry, Orissa University of Agriculture and Technology, Bhubaneswar - 751 003, Odisha, India; 2 Department of Veterinary Anatomy and Histology, College of Veterinary Sciences and Animal Husbandry, Orissa University of Agriculture and Technology, Bhubaneswar - 751 003, Odisha, India; 3 Department of Veterinary Pathology, College of Veterinary Sciences and Animal Husbandry, Orissa University of Agriculture and Technology, Bhubaneswar - 751 003, Odisha, India; 4 Department of Livestock Products Technology, College of Veterinary Sciences and Animal Husbandry, Orissa University of Agriculture and Technology, Bhubaneswar - 751 003, Odisha, India

**Keywords:** benign prostatic hyperplasia, dogs, hemato-biochemical study, urinalysis

## Abstract

**Aim:**

The study was designed to evaluate the hemato-biochemical alterations, urinalysis along with histomorphological and histological changes of prostate glands in dogs affected with benign prostatic hyperplasia (BPH) in and around Bhubaneswar, Odisha, India.

**Materials and Methods:**

In toto, 445 dogs presented to the Teaching Veterinary Clinical Complex of the College of Veterinary Sciences and Animal Husbandry, one Government Veterinary Hospital and two pet clinics in and around Bhubaneswar screened for the presence of BPH. Most of the 57 dogs were 6 years and above as reported by the owners. Only 57 dogs found positive for BPH basing on the presence of typical clinical signs subjected for a detailed hemato-biochemical study. Most of the 57 dogs were 6 years and above as reported by the owners. Routine and microscopic urinalyses were done as per the routine procedure. Histomorphological evaluations of prostate glands were done through manual rectal palpation. Histological examinations of prostate tissue sections of two dead dogs were conducted with routine hematoxylin and eosin stain.

**Results:**

The study revealed about 12.8% (57/445) of dogs was suffering from BPH. Typical clinical signs - such as passing small thin tape-shaped feces, holding tail away from backward, tenesmus, and straining during urination and defecation - were seen in most of the cases. Urine samples of affected dogs were positive for glucose, occult blood, and protein. A significant decrease in lymphocytes and increase in eosinophil counts in dogs with BPH was recorded. Serum biochemical analysis showed a nonsignificant increase in creatinine and blood urea nitrogen with a significant decrease in total protein, albumin, globulin, A:G ratio. Histology of prostate glands collected during postmortem was characterized by fibrosis of prostate gland, and hyperplasia of the acinar epithelium.

**Conclusions:**

High rate of the prevalence of BPH in dogs poses an alarming condition which if diagnosed at an early stage can certainly prolong the longevity of the dogs.

## Introduction

Prostate disorders are commonly noticed in dogs particularly in un-neutered intact and middle-aged male dogs [1]. When an un-neutered male dog reaches 8 years of age, it has a >80% chance of developing prostate diseases [2]. Older intact male dogs of all breeds are preferably affected with prostate disorders [3]. Middle and big sized breeds are prone to development of prostatic disease, with Doberman pinscher and German shepherd being affected more frequently than other breeds [4]. Types of prostate abnormalities seen in dogs include benign prostatic hyperplasia (BPH), cysts, abscesses, acute and chronic infections and neoplasia [4-6]. Neutering is often recommended as a part of therapy regardless of the type of prostatic disease presents [7].

It is important to determine early whether the dog has an easily treatable condition or something more serious like prostate cancer [8,9]. Dogs suffering from prostatic disease show the clinical symptoms of straining to urinate, frequent voiding of small amount of urine, blood tinged urine, dripping of blood from the penis, constipation and straining to defecate, lethargy, fever, holding tails slightly away from back, weight loss and passing ribbon-shaped feces [10].

The present research work was designed to study the clinical signs, hemato-biochemical, urinalysis, histomorphological, and histological aspects of prostate tissue of dogs suffering from BPH.

## Materials and Methods

### Ethical approval

The experiment was carried out according to the national regulations on animal welfare and in Institutional Animal Ethical Committee.

### Place of the study

This study was undertaken in the Department of Veterinary Clinical Medicine, College of Veterinary Sciences and Animal Husbandry, Odisha University of Agriculture and Technology (OUAT), Bhubaneswar, during the period from October 2010 to September 2012.

### Sources of dogs

The dogs brought/referred to the Teaching Veterinary Clinical Complex, College of Veterinary Sciences and Animal Husbandry, OUAT; Government Veterinary Hospitals and private pet clinics in and around Bhubaneswar city were the sources of dogs for the present study. In toto, 445 dogs examined for the presence of BPH.

### Selection of dogs

Dogs with one or more complaints/history of straining during urination and defecation, urinary incontinence, holding tail slightly away from back, dribbling blood from the penis during urination, lethargy, passing small thin tape-shaped feces, and in-coordination in movement particularly in hind limbs were screened for BPH.

### Manual rectal examination

The dogs exhibiting the above clinical signs were subjected to rectal palpation for confirmation of BPH. The rectal palpation was done manually with hands/digits by the experienced clinician with assisted caudodorsal abdominal pressure, shifting the gland to the pelvic inlet which was easiest to achieve on a standing patient. On the medium sized dog, the prostate was physiologically the size of a walnut, with a smooth surface, solid consistency, free, isothermal and does not cause pain to the animal during examination [1,7]. Symmetry was evaluated after the central sulcus was identified on the dorsal surface of the gland. From this sulcus, both right and left lobes of the same size originate. In giant breed dogs, the prostate is often barely palpable and diagnostic ultrasound may be the only dependable method of evaluation of size and inner structure of the gland. Anyway, prostatic rectal palpation is considered the basic non-invasive method and should be utilized as a screening method whenever possible [1].

### Hemato-biochemical analysis

Blood collected aseptically with ethylenediaminetetraacetic acid anticoagulant subjected for detail estimation of hemoglobin (Hb), total leukocyte count (TLC), packed cell volume (PCV), total erythrocyte count (TEC), and differential count (DC). Serum collected was analyzed for various parameters such as mean serum glucose, creatinine, blood urea nitrogen (BUN), total protein, albumin, globulin, A:G ratio and potassium by spectrophotometer using commercial reagent kits.

### Statistical analysis

Statistical analysis was performed using Microsoft Excel spreadsheet.

## Results and Discussion

### Prevalence of prostate disorders (BPH)

This investigation revealed that out of 445 dogs examined, 12.8% (57/445) of dogs were suffering from BPH. Similar reports regarding a high prevalence of this disease have also been recorded by the findings of Polisca *et al*. [4], Jayaraja *et al*. [5], Mukaratirwa and Chitura [8]. This high prevalence rate of BPH may be accounted for disproportionate sample size with respect to intact males, avoidance of mating, neutered males, more secretion of testosterone, rapidly changing estrogen/androgen ratio when estrogen predominates, and region-based management practices [2,6].

### Breed

Breed-wise analysis showed that the Doberman, German Shepherd, and Labrador breeds were more susceptible to BPH than the rest of the breeds included in the study. Other related studies have reported that the prevalence of BPH seemed to be higher in large-sized breed dogs such as GSD and Doberman which corroborates with this finding [4,10].

### Clinical signs

The results of the study showed that passing small thin tape-shaped feces was one of the common clinical sign found in all the 57 (100.0%) dogs suffering from BPH. The other clinical signs recorded were straining during urination and defecation, urinary incontinence, tenesmus, hematuria/blood dribbling from penis, holding tail away from backward, weakness, body weight loss, inappetence, and hind limb paralysis in 91.2, 87.7, 61.4, 45.6, 38.5, 33.3, 26.3, 22.8, and 8.7%, respectively. This characteristic clinical sign owes to increased size of prostate gland which compresses the colon and interferes with defecation causing rectal tenesmus and constipation. The enlarged prostate compresses the rectum for forming ribbonlike stools. Prostitis is the most common complication of BPH in dogs due to secondary bacterial infections [9-12].

### Histomorphological study

Diagnosis of BPH is based on the presence of typical clinical signs and on detecting a uniform prostatic enlargement by palpation, radiography, and/or ultrasonography. The caudal/dorsal aspect of the prostate in most dogs can be palpated via the rectum, although the position of the prostate in the caudal abdomen depends on bladder distention, age, and disease. On manual rectal palpation, the hypertrophied prostate is found to be enlarged but symmetric, soft with a smooth contour, movable, and painless [1,6].

Histomorphological changes of the prostate gland in affected dogs assessed by the manual palpation of prostate gland through rectum showed about 57 (100%) affected dogs were having bigger than normal size (walnut) of prostate gland. The other characters recorded in this study described in Table-1. It was found that more than 70% of the affected dogs exhibited bigger size, asymmetric, rough, soft, pain on palpation, and hyperthermic prostate gland except unmoveable prostate were detected in 68.4% of dogs. Pain on palpation may be due to increased pressure on the local nerve endings by the enlarged gland and enlargement is due to hyperplasia of acinar epithelium [7-10].

**Table-1:** Morphology of prostate through rectal examinations in dogs with BPH.

Physical characters	Healthy dogs (n=10)	Dogs with BPH (n=57)	Percentage of abnormality
Size	Walnut	Bigger than walnut size	57 (100.0)
Symmetry	Symmetric	Asymmetric	41 (71.9)
Surface contour	Smooth	Rough	43 (75.4)
Consistency	Solid	Soft	54 (94.7)
Movability	Moveable freely	Unmovable	39 (68.4)
Pain on palpation	Painless	Pain on palpation	55 (96.5)
Isothermic	Present	Hyperthermic	40 (70.1)

Figures in parentheses indicate percentage. BPH=Benign prostatic hyperplasia

### Urinalysis

Analysis of urine with respect to color, turbidity, specific gravity, urine pH, glucose, ketone, bilirubin, occult blood, protein, bacteria, and crystals in both healthy (n=10) as well as dogs with BPH (n=57) have been presented in Table-2. The color of the urine in both cases of healthy and BPH affected dogs were found to be yellow without any variations. None of the urine sample collected from the healthy dog showed the presence of glucose, ketone, occult blood and presence of any bacteria whereas the samples collected from the affected dogs were positive for glucose and occult blood. Increased Specific gravity, presence of protein, glucose and occult blood in affected dogs might be related to kidney dysfunction or nephritis. Secondary bacterial infection causing prostaitis is a contributing factor for acidic urine and prostatic fluid as well as a leading cause for forming nidus resulting presence of crystals in urine [6,10,11].

**Table-2:** Urinalysis of dogs exhibiting BPH.

Parameters	Healthy geriatric dogs (n=10)	Dogs with BPH (n=57)
Color	Yellow	Yellow
Turbidity	Clear	Mild turbid
Urine specific gravity (mean±SE)	1.015-1.045	1.013-1.041
Urine and/or prostatic fluid pH (mean±SE)	5.0-7.1	6.1-6.5
Glucose	Negative	Positive
Ketone	Negative	Negative
Bilirubin	Trace to 1+	Trace to 1+
Occult blood	Negative	Positive
Protein	Trace	More
Bacteria (per high power field)	Negative	Negative
Crystals (per high power field)	Variable	Variable

BPH=Benign prostatic hyperplasia, SE=Standard error

### Hematological study

The mean±standard error values of various hematological parameters with respect to Hb, PCV, TEC, TLC, and DC were depicted in Table-3. Statistical analysis revealed a significant decrease in lymphocytes and significant increase in eosinophil counts in dogs with BPH which might be due to secondary bacterial infection of chronic in nature. The cause of slight reduction of Hb and PCV values may be due to hematuria [8]. This may also be due to decreased bone marrow production, splenomegaly, and decreased erythrocyte production. It may also occur due to deficiency of vitamins like copper and zinc in old dogs. Lower value may also be encountered due to urinary obstruction resulting chronic kidney disease that results in decreased erythropoietin production and decrease capacity of bone marrow to produce red blood cell, which ultimately results in lower TEC production [5-8]. The elevation in eosinophil percentage might be due to decomposition of body protein.

**Table-3:** Mean±SE of hematological values of dogs with BPH.

Parameters	Mean±SE	

	Healthy dogs (n=10)	Dogs with BPH (n=57)
Hb (g/dl)	11.58±0.32 (10.40-13.40)	11.21±0.45 (10.92-11.63)
PCV (%)	34.88±0.81 (31.0-39.0)	34.34±3.71 (31.20-38.18)
TEC (n×10/µl)	6.86±0.25 (5.84-8.79)	6.76±0.25 (6.20-7.01)
TLC (n×10^3^≥/µl)	10.39±0.51 (7.78-11.98)	10.61±0.86 (9.25-11.37)
Differential counts: Neutrophils (%)	66.85±1.61 (64.0-75.0)	66.90±4.25 (61.8-72.9)
Eosinophils (%)	5.41±0.39 (3.0-6.0)	8.90±0.41 (7.8-9.64)
Basophils (%)	-	-
Lymphocytes (%)	22.68±1.25 (16.0-29.0)	18.70±0.89 (16.8-20.47)
Monocytes (%)	4.98±0.45 (3.0-6.0)	5.50±0.22 (4.99-5.92)

Figures in parentheses indicate minimum and maximum values. BPH=Benign prostatic hyperplasia, SE=Standard error

### Biochemical parameters

Mean serum glucose, creatinine, BUN, total protein, albumin, globulin, A:G ratio and potassium of affected (n=57) as well as healthy dogs (n=10) were analyzed and recorded in Table-4. Statistical analysis of the data obtained in this study indicated nonsignificant increase in creatinine and BUN and a significant decrease in total protein, albumin, globulin, A:G ratio, potassium and minor decrease level of glucose. The mean values of total protein level in clinical cases of BPH (43.1±5.2 g/L) were found to be significantly lower, indicating the possible presence of hypoproteinemic status which might be due to inappetence and weakness. In dogs with BPH, the mean albumin value was significantly decreased which was also reflected in the reduced A:G compared with the values of apparently healthy ones. This observation is in accordance with the findings [5,13] has indicated that the significantly decreased level of albumin and nonsignificant decreased level of globulin in dogs might be due to inappetence, weakness and body weight loss. The mild increased level of BUN is believed to be characterized by increased catabolism due to inappetence and progression of kidney infections [6,14].

**Table-4:** Mean±SE of biochemical values of dogs with BPH.

Parameters	Mean±SE	

	Healthy dogs (n=10)	Dogs showing BPH (n=57)
Blood glucose (mmol/L)	5.05±0.67 (3.61-6.55)	4.99±0.32 (4.12-5.25)
Serum creatinine (µmol/L)	60.06±5.21 (49.1-72.6)	62.5±4.8 (55.9-69.1)
BUN (mmol/L)	4.25±0.74 (3.65-5.39)	5.79±0.54 (4.99-6.13)
Serum total protein (g/L)	61.2±5.6 (54.5-72.8)	43.1±5.2 (39.2-50.1)
Serum albulin (g/L)	29.3±1.8 (26.1-33.2)	13.3±1.2 (11.9-14.8)
Serum globulin (g/L)	28.4±3.4 (24.1-30.6)	24.9±3.1 (20.45-27.9)
A:G	0.9±0.12 (0.7-1.1)	0.67±0.05 (0.51-0.69)
Serum potassium (mmol/L)	4.68±0.4 (4.32-5.46)	3.94±0.18 (3.64-4.35)

Figures in parentheses indicate minimum and maximum values. BUN=Blood urea nitrogen, BPH=Benign prostatic hyperplasia, SE=Standard error

### Histological study

**Figure-1:**
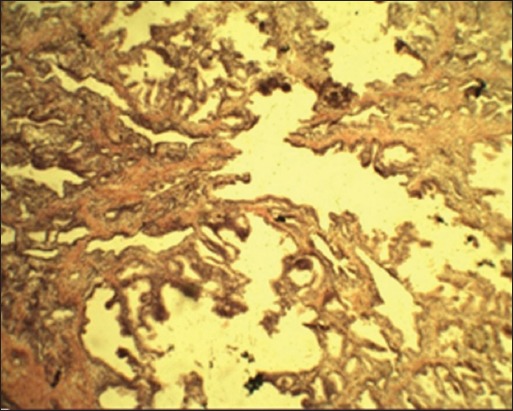
Photomicrograph of prostate gland showing prominent cystic acini (H and E, 100×).

Routine histological examinations of the prostate gland of dogs affected with BPH showed fibrosis, prominent cystic acini (Figure-1), hyperplasia of acinar epithelium, and concentric laminated concretions of secretory product in acini which corroborated with the reports of earlier workers [1,4,15].

## Conclusion

There was an overall prevalence of 12.8% of BPH. Typical clinical signs such as passing small thin tape-shaped feces, holding tail away from backward, tenesmus and straining during urination and defecation were characteristics in most of the cases. Urine samples of affected dogs were positive for glucose, occult blood, and protein. There was a significant decrease in lymphocytes and increase in eosinophil counts in dogs with BPH. Serum biochemical analysis showed a nonsignificant increase in creatinine and BUN with a significant decrease in total protein, albumin, globulin, A:G ratio. Histology of prostate glands collected during post-mortem was characterized by fibrosis of prostate gland, and hyperplasia of acinar epithelium.

## Authors’ Contributions

MRD carried out the experiment and drafted the final manuscript. RCP and RKD designed the experiment and guided during the experiment. PKR and BPM collected the scientific literatures and prepared the first draft of the manuscript. All authors read and approved the final manuscript.
